# Portugal and It's Drugs Policy - What Changed?

**DOI:** 10.1192/bjo.2022.359

**Published:** 2022-06-20

**Authors:** Mariana Macedo Vieira, Ana Raquel Faria

**Affiliations:** 1 Greater Manchester Mental Health NHS Foundation Trust, Manchester, United Kingdom; 2 Hospital de Braga, EPE, Braga, Portugal

## Abstract

**Aims:**

The authors intend to briefly review the literature regarding the progress of substance misuse rates and harms, the support available and reflect on the current national situation. We aim to better understand the changes in policies and services, and their impact to see what can be learned from the Portuguese experience.

**Methods:**

A narrative literature review was carried out by the authors using the keywords “addiction” “drugs policy” “Portugal” “drug use”. The authors declare no conflicts of interest.

**Results:**

The end of the dictatorship in 1974, the reopening of borders, and the return of white Portuguese from former colonies, were all associated with a dramatic increase in substance misuse in Portugal. In the 1990's it is estimated that 0.5%-1% of the population was using heroin at the time, with extremely high rates of HIV and Hepatitis in intravenous drug users.

At the start of this period, healthcare services were poorly organized, resources for substance misuse services were limited, legislation was punitive, and there was a general understanding that drug addiction was a consequence of a moral failing. A change in paradigm occurred in the late 1990's and new legislation introduced in 2001, which along with the growth in services to support substance misusers dramatically reduced the rates and negative outcomes of substance misuse.

Of note, new HIV diagnoses due to injecting and overdose rates dropped significantly in the XXI century and decriminalization did not lead to an overall increase in substance misuse.

**Conclusion:**

The pivotal shift in the understanding of the nature of addiction as an illness lead to a profound change in interventions to mitigate this overwhelming problem that affected Portugal by the end of the XX century. We hope that by sharing this experience this will improve interventions around the world to support substance misusers and public health.

